# Fetal Antecedents of Cardiovascular Disease

**DOI:** 10.1161/JAHA.118.010507

**Published:** 2018-09-26

**Authors:** Justin R. Ryder

**Affiliations:** ^1^ University of Minnesota Medical School Minneapolis MN; ^2^ Center for Pediatric Obesity Medicine University of Minnesota Minneapolis MN

**Keywords:** Editorials, preeclampsia/pregnancy, pregnancy, pregnancy and postpartum, Preeclampsia, Atherosclerosis, Coronary Artery Disease, Cardiovascular Disease, Pregnancy

## Introduction

The origins of cardiovascular disease (CVD) are firmly placed in childhood and are progressive during the lifecycle. Seminal autopsy studies from Strong and McGill in the 1960s demonstrated clearly that the earliest visual manifestations of the atherosclerotic CVD (eg, fatty streaks) were often present in children aged 10 to 19 years and even observable in children as young as 1 year.^1^, ^2^, ^3^ These important early observations laid the foundation for examining the origins of CVD development and other potential risk factors. Maternal contributors, such as smoking, physical inactivity, obesity, and dietary intake, all have been linked with increased development of CVD risk factors in offspring.^4^, ^5^, ^6^ Mothers with high‐risk cardiovascular conditions, such as hypercholesterolemia and hypertension, are more likely to have offspring with more advanced visual atherosclerotic manifestations.^7^, ^8^ Additionally, several CVD risk factors and surrogate markers of subclinical CVD are, at least in part, heritable from parent to child.^6^, ^9^ The heritable contribution can largely be attributed to genetics and, to a lesser degree, shared environmental exposures. However, the degree or extent of tracking of CVD risk factors from mother to offspring is largely unknown, and limited data are available using robust measures of the microvascular and macrovascular structure and function in both mother and offspring.

In this issue of the *Journal of the American Heart Association* (*JAHA*), Benschop and colleagues, using data in 5624 mother‐offspring pairs for the Generation R Study, examined the association between maternal and childhood CVD risk factors at the ages of 6 and 9 years.^10^ In this large sample of mother‐offspring pairs, the authors examined the association of multiple microvascular (central retinal arterial and venular caliber) and macrovascular (blood pressure, left atrial and aortic root diameter, left ventricular mass, fractional shortening, and pulse wave velocity) variables in mothers and children at the age of 6 years (only blood pressure measured at the age of 9 years). Importantly, the analysis accounts for many relevant exposures (confounders), such as maternal education level, ethnicity, smoking, sex of offspring, prepregnancy body mass index, and maternal blood pressure. With and without accounting confounders, all microvascular and macrovascular variables were highly associated between mother and offspring aged 6 and 9 years after pregnancy. Additionally, the authors conduced analysis removing mothers with pregnancy complications known to be associated with high‐risk offspring (eg, preeclampsia, small for gestational age, and preterm birth) and presented the same findings. The comprehensive CVD risk profile examined and tracked over many years leaves little doubt that adverse maternal CVD risk factors are strongly associated with a similar adverse CVD risk profile in their offspring.

These data add support to primary prevention efforts for CVD that are likely best placed in early childhood, particularly for offspring of mothers who have high CVD risk factors before and during pregnancy. Identifying children of mothers with high CVD risk factors may serve as an ideal target population for these prevention efforts. Moreover, these data also suggest that primordial prevention of CVD likely needs to occur in mothers before or during pregnancy because the development of risk factors in mothers is more likely to produce an adverse profile in children. Interventions toward planned or expecting mothers may be an ideal target to prevent and delay the earliest manifestations of the CVD process, which, in turn, may delay the onset of CVD morbidity and mortality for many years in offspring.

## Disclosures

None.
